# Complete Cerebrospinal Fluid Response to T-DM1 in HER2 Positive Metastatic Breast Cancer: A Case Report

**DOI:** 10.3390/life13030756

**Published:** 2023-03-10

**Authors:** Francesca Zacchi, Elena Giontella, Alessia Nottegar, Elena Fiorio

**Affiliations:** 1 Section of Oncology, Department of Medicine, University and Hospital Trust of Verona, 37126 Verona, Italy; francyzacchi@gmail.com (F.Z.); elena.fiorio@aovr.veneto.it (E.F.); 2 Department of Pathology and Diagnostics, University and Hospital Trust of Verona, 37126 Verona, Italy; alessia.nottegar@aovr.veneto.it

**Keywords:** breast cancer, leptomeningeal carcinomatosis, leptomeningeal metastasis, anti-HER2 therapy, human epidermal growth factor receptor 2, cerebrospinal fluid, T-DM1 therapy

## Abstract

Leptomeningeal carcinomatosis is a rare but serious consequence of pre-existing tumors, such as breast, lung, and gastrointestinal carcinomas. Further, leptomeningeal carcinomatosis is more frequently diagnosed with breast cancers, if only because breast cancers are diagnosed far more often than any other carcinomas. In this paper, we present the case of a leptomeningeal carcinomatosis patient who experienced complete remission following therapy targeted at the Her-2 (human epidermal growth factor receptor 2-positive) receptor. This patient’s diagnosis was complicated by the fact that brain and column MRI imaging were clear, but analysis of the cerebrospinal fluid led to the conclusion of leptomeningeal carcinomatosis. The tests were requested because the patient, under chemotherapy for advanced breast cancer at the time, reported some neurological symptoms. Following the diagnosis of leptomeningeal carcinomatosis and subsequent T-DM1 Her-2 receptor therapy, the patient showed a complete response to leptomeningeal carcinomatosis within 30 days and survived for another 16 months. This case offers compelling evidence that the effect TDM1 Her-2 receptor therapy has on a patient’s remission and long-term survivability is considerably better than other therapies for similar pre-existing conditions diagnosed with leptomeningeal carcinomatosis. Further prospective studies should confirm these findings.

## 1. Introduction

Leptomeningeal carcinomatosis (LC) is a rare, but very serious, condition and is most often diagnosed in breast cancer (BC) patients, occurring in a total of 5% of BC patients. This correlation is related to the fact that BC is diagnosed far more frequently than any other cancer. 

LC is characterized by a severe impact on morbidity and mortality incidence and retains a dismal prognosis, even in patients treated with multi-modal, aggressive treatments. 

In fact, after a diagnosis of LC, the prognosis remains poor, with a median survival of a few months for patients receiving state-of-the-art treatment. LC leads to a devastating prognosis; however, disease presentation and prognostic factors are uncertain [1]. Concerning clinical features, LC can present itself with a large variety of symptoms making the diagnosis complicated; symptoms of LC arise according to the area of the central nervous system (CNS) involved with neoplastic cells.

Symptoms may be subtle and unspecific, especially in the initial phases of the disease, and this may often lead to a delay in diagnosis. Headache is often one of the first clinical presentations of LC (present in >80% of patients at diagnosis). Other signs and symptoms include nausea, vomiting, radicular pain, cranial nerve deficits, visual disturbances, hearing loss, seizures, and cauda equina syndrome. An uncommon possible clinical presentation is new onset of a psychiatric disorder. In a minority of cases, LC may be diagnosed incidentally in asymptomatic patients.

After suspicion of LC, today there is no consensus on the diagnosis of LC, which often requires a combination of neurological assessment, imaging, and CSF analysis. The diagnostic work-up generally requires CSF cytology and radiological imaging with brain and column MRI, which are two tools with low sensitivity, particularly in the early stages of LC [2].

To overcome the problem of low sensitivity, more sensitive techniques are being studied. The lack of a gold standard for LC diagnosis also contributes to the high heterogeneity of diagnosis in patients enrolled in dedicated studies [3]. Major clinical societies have tried to establish guidelines for a uniform approach to diagnosing LC. Regarding the diagnosis of LC from solid cancers, the joint European Association of Neuro-Oncology(EANO)—European Society for Medical Oncology (ESMO) guidelines recommend MRI of the brain and spine in conjunction with CSF cytologic analysis. Similarly, the Leptomeningeal Assessment in Neuro-Oncology (LANO) working group (a part of the Response Assessment in Neuro-Oncology [RANO] group) recommends the presence of a combination of suggestive symptoms, brain and spine MRIs, and CSF cytology to establish a diagnosis of LC. However, particularly at early stages, MRI findings can be non-diagnostic and definitive findings may therefore appear only in later stages. Likewise, while positive CSF cytology represents the gold standard for diagnosing LC, its initial sensitivity is modest, and its sensitivity can increase to about 90% in a repeated CSF sample. Moreover, once the diagnosis is established, MRI and CSF cytology are insensitive to measuring treatment response.

Concerning LC, prognostic factors are also uncertain. Among the various factors studied, the main factors associated with prognosis are performance status at diagnosis, CFS protein level, and triple-negative subtype. Studies in the literature show a possible correlation between BC histology and development of LC; invasive lobular carcinoma seems to have a higher propensity to metastasize in the leptomeninges when compared to ductal carcinoma, with a prevalence two times higher among patients with leptomeningeal disease than among the whole BC population [4].

The intrinsic subtype most commonly associated with LC is triple-negative breast cancer (TNBC). Although human epidermal growth factor receptor 2-positive (HER2+) BC shows central nervous system (CNS) tropism in the form of metastasis to the brain parenchyma, LC is not a common manifestation of this intrinsic subtype. HER-2 receptor tyrosine kinase is one of the most studied biomarkers in BC. Amplification or overexpression of HER-2 is detected in almost 25% of primary BC cases [5] and it has a poor prognostic value but a predictive value of response to different types of target drugs. Trastuzumab, a recombinant humanized monoclonal antibody to Her-2, has shown clinical activity in combination with chemotherapy in the metastatic and adjuvant setting. Moreover, multiple new Her-2–directed therapeutics have been developed or are under testing.

The relationship between Her-2 overexpression in primary breast tumors and the development of brain metastasis (either in parenchyma or leptomeninges) has been the subject of considerable investigations. There are multiple possible causes of increased brain metastases among Her2–positive BC patients. Her-2 overexpression may affect the development of breast cancer and accelerate brain progression; poor penetration of the blood–brain barrier (BBB) by trastuzumab may render the brain a ‘‘sanctuary’’ site for metastases. The increased life span of patients receiving trastuzumab therapy may permit brain metastases to become evident [6]. From the literature, Her2+ BC patients with LC having received systemic Her2-targeted therapy seem to be a factor with independent prognostication with better prognosis [7]. To date, there are no specific recommendations regarding systemic treatment for LC in breast cancer.

Treatment recommendations are typically rather individualized because they depend on various factors, such as the site of primary tumor, brain and meningeal disease pattern (e.g., the absence or presence of concurrent systemic or solid brain metastasis), and the absence or presence of tumor cells in CSF [8]. Different drugs have been tested to treat BC with LC, including different chemotherapy agents and hormonal agents, which have been reported to show occasional response to LC.

In human epidermal growth receptor 2 positive (HER2+) BC with LC, the European guidelines suggest HER2-targeted treatment in combination with chemotherapy. However, these recommendations are based on uncontrolled case series and expert opinions rather than data from randomized clinical trials [9]. Moreover, the treatment of leptomeningeal carcinomatosis is challenging due to the presence of the blood–brain barrier; numerous systemic therapies do not readily penetrate into the site of leptomeningeal disease and have been ineffective.

Although the lack of high-level evidence about the impact of the blood–brain barrier (BBB), as well as the efficacy of anti-cancer agents on the brain, the understanding of the activity of systemic treatments with intrathecal impact is rapidly evolving. Novel anti-HER2 agents, such as tucatinib, trastuzumab emtansine, trastuzumab deruxtecan, and neratinib, have shown intracranial efficacy. Current research efforts are ongoing not only to clarify the activity of existing treatments on the CNS, but also to develop new drugs and innovative multi-modality approaches [10].

Moreover, intrathecal administration of monoclonal antibodies, such as Trastuzumab, has been investigated. It shows better survival outcomes, but more data and experience are necessary before intrathecal administration can be considered standard. At the moment, it remains an investigational drug [11].

In this paper, we present the case of a patient with HER2-positive metastatic BC with LC diagnosed only in the cerebrospinal fluid (CSF), with both brain and column negative MRI, with a long response to anti-HER2 therapy with Trastuzumab emtansine (TDM-1) and with a complete response on LC in a very short time.

## 2. Case Report

A 50-year-old woman, in a good clinical condition, was diagnosed with breast cancer in 2014. A staging with breast magnetic resonance imaging (MRI) with a contrast and iodine-based contrast total body CT scan were performed and the results showed that the tumor was localized only in the breast; therefore, in 2014, the woman underwent a mastectomy and axillary lymph node dissection. The final stage was an invasive ductal carcinoma pT2 (3 cm) pN1a (3/13), M0, G2, Ki67 8% with overexpressed hormonal receptors (ER 100%, PgR 97%), while HER-2 resulted not expressed (the amplification in IHC was 1+ and FISH was not performed). After surgery, we decided to propose adjuvant chemotherapy to the patient according to the FEC scheme for 3 cycles and Paclitaxel weekly for 12 cycles, which concluded on 27 April 2015. This was followed by adjuvant hormone therapy with tamoxifen until March 2020.

Unfortunately, in February 2020, during the follow-up, a single bone recurrence in the left acetabulum was found on the restaging CT scan and the patient came into our oncologic day hospital.

We started immediately with bisphosphonate therapy (scheduled 4 weekly), but we needed a biopsy of the bone lesion for the retyping of the disease, given the time elapsed since the previous diagnosis of breast cancer.

Bone lesion biopsy was conducted and resulted in metastasis of the primary tumor with a hormonal profile (ER 100%, PgR not evaluated), Ki67 9%, and, at this time, an expression of HER2 (amplification 3+ in IHC). Therefore, given these characteristics, in March 2020, the patient started first-line therapy with Trastuzumab (6 mg/Kg q21 days intravenously) plus Pertuzumab (420 mg q21 days intravenously) plus Paclitaxel (80 mg/mq q7 days intravenously) and bisphosphonate therapy with Zometa. Because of important neurological toxicities (sensory neuropathy G3 according to CTCAE v.5), chemotherapy with Paclitaxel was stopped after 13 administrations, but maintenance therapy with Trastuzumab plus Pertuzumab plus bisphosphonate therapy with Zometa was continued and an anti-hormonal therapy with Faslodex was started. Given the persistence of only a single stable metastatic bone lesion, in August 2020, the patient underwent curative intent radiotherapy for bone metastasis with a clinical benefit. At the subsequent restaging performed three months after radiotherapy, the pelvic MRI showed a radiological partial response, but the 18F-FDG PET/TC showed evidence of hypermetabolism (SUV 8.9) at the level of the known osteolytic bone metastasis. As a result, in February 2021, we decided to subject the patient to electrochemotherapy (ECT) at the level of the left acetabular metastasis, with curative intent, at a dedicated hospital.

Unfortunately, in March 2021, the patient started to present episodes of confusion and balance disturbance so she was recovered, but the brain MRI, performed during the recovery, presented a negative result for brain metastases.

**Figure 1:**
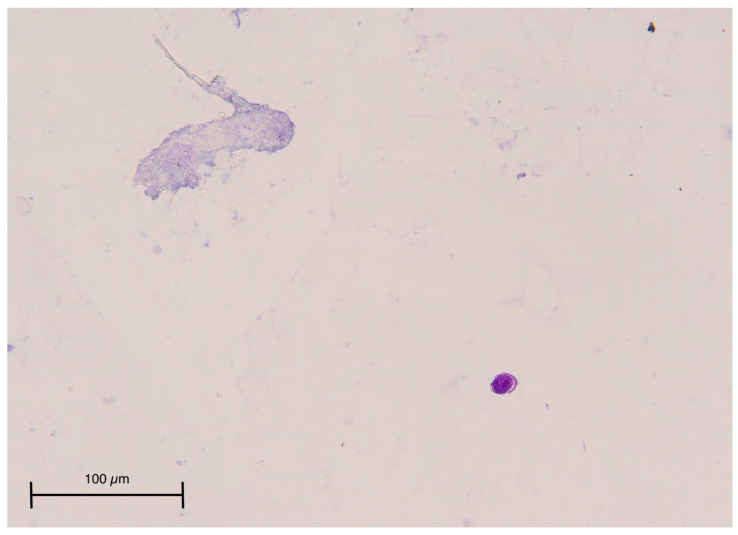
Cerebrospinal fluid (H&E, original magnification 20×). Cytological examination of the cerebrospinal fluid revealed scattered epithelial cells that were round, large, and highly variable in size nuclei. On immunohistochemical analysis, these cells demonstrated a strong positivity for GATA3, suggestive of mammary origin.

For the persistence of neurological symptoms, such as tinnitus and balance disturbance, we performed CSF cytology, which revealed neoplastic cells with strong positivity after immunohistochemistry for GATA3 that suggested mammary origin, so the pathologist suspected LC [Figure 1].

**Figure 2:**
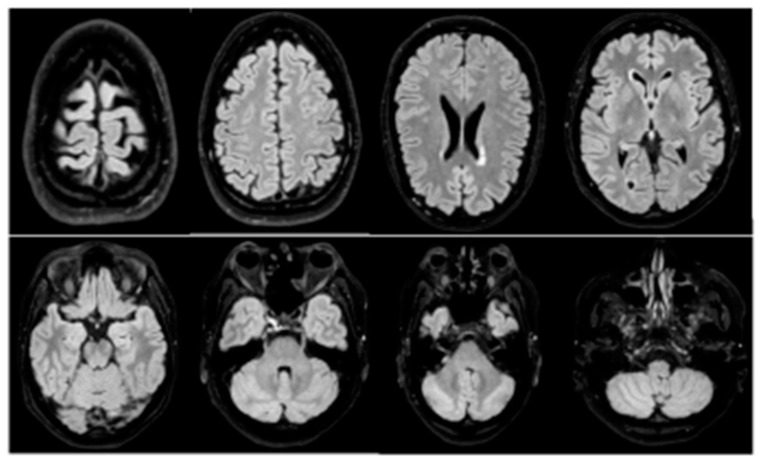
Gadolinium-based brain MRI: negative for brain metastasis or meningeal carcinomatosis. In particular, no appearance of signal alterations in the cerebral parenchyma both in the supratentorial and subtentorial sites. No areas of diffusion restriction to refer to recent ischemic lesions. After administration of gadolinium, no pathological enhancement of the cerebral parenchyma or meninges. Unchanged morphology and dimensions of the ventricular system. Structures of the median line in axis.

At the time of CSF analysis, a brain MRI was performed, which showed a negative result for brain metastases or meningeal carcinosis [Figure 2].

Therefore, on the basis of positive liquor analysis, in May 2021, the patient started second-line therapy with TDM-1 (Trastuzumab/Emtansine), with a complete pathological response on LC. In fact, in June 2021, the patient underwent a subsequent lumbar puncture that showed a negative result for neoplastic cells. Furthermore, a new brain and column MRI showed a negative result for metastases. The pathological response, in addition to being complete, was also lasting. In fact, last-stage brain MRI, after sixteen months from the beginning of TDM-1 therapy, did not show pathological disease and the patient did not present neurological symptoms; moreover, the pelvis MRI showed the dimensional stability of the bone metastasis in the left hip, with a greater sclerotic component, an index of a likely response to therapy. Additionally, FDG PET showed mild hypermetabolism of left hip metastasis (SUV max 4.2 versus 8.9 before the beginning of TDM-1 therapy). Therefore, given the radiological response on the bone and, above all, the disappearance of malignant cells in CSF, we decided to continue anti-HER2 therapy TDM-1 in the absence of adverse events and in combination with bisphosphonate therapy.

## 3. Discussion

In patients with metastatic breast cancer, the presence of LC is a rare condition and a devastating disease that generally determines a poor prognosis. The different biology of breast cancer’s subtypes seems to have a role in the incidence of LC. In fact, patients with HER2+ BC seem to be more neurotropic than other breast cancer subtypes, with more evident spreading in the brain [6]. Furthermore, in BC patients with Her-2–positive tumors, brain metastases also occur during a responsive or stable disease at other sites.

From the literature data, after diagnosis of LC in BC patients, overall survival (OS) is only a few months with personalized treatment plans. Moreover, LC is likely to cause neurological damage. Not having neurological symptoms and receiving radiotherapy (RT) at LC diagnosis seem to be associated with prolonged OS. Survival seemed to also be prolonged with multi-modality treatment, which included targeted therapy, intrathecal chemotherapy, and RT to the LC sites [12].

An important issue is that the diagnosis of LC after neurological symptoms can be difficult, especially in the early stage. There is no gold standard for LC diagnosis; MRI and cerebrospinal fluid cytology are the most frequently used modalities, despite the low accuracy [13].

In our case, the diagnosis was very difficult because the patient had either a negative brain or total column MRI, and the LC diagnosis was given by CSF cytology alone. Another important feature of our case is that, despite a lot of findings that seem to be associated with a worse prognosis, such as the absence of the use of RT on LC (because there was no evidence of LC on radiological imaging) and despite the presence of neurological symptoms, the OS reported in the literature is much longer than the median OS of HER2+ metastatic BC with LC [14].

Diagnosis and treatment decisions for patients with leptomeningeal metastasis from solid tumors vary widely across Europe. In contrast to the limited data in the literature about HER2-target therapy for LC, which is often based on case reports or case series rather than data from randomized clinical trials, our case suggests that target therapy with TDM-1 shows intracranial effect.

Therefore, it is important to improve research on current target drugs and their efficacy on LC, possibly in randomized prospective clinical trials. Standardization of diagnosis and evaluation tools, as well as controlled studies to improve the level of evidence for all therapeutic approaches to LM, are required. Optimal management of LM also requires multidisciplinary care and diagnosis and treatment strategies should ideally be developed in tumor boards.

It is important to develop innovative drugs to improve the multi-modality approach and, above all, to carry out further research on different subtypes of BC to develop more personalized therapy for each patient.

## 4. Conclusions

In our case, there are several particular aspects. First of all, the clinical presentation is uncommon. In fact, LC was suspected when the patient began to show unusual symptoms, such as the manifestation of tinnitus and confusion, as well as balance disturbances. The other very particular aspect is linked to the fact that, following the suspicion of meningeal carcinomatosis or brain metastases, given the symptoms, a diagnostic work-up was started, which highlighted a positive cerebrospinal fluid analysis with totally negative brain and column imaging.

Ultimately, the absolutely most extraordinary aspect is that, in consideration of the expression of HER2, systemic therapy with TDM-1 was evaluated and this drug led to a complete remission of leptomeningeal carcinomatosis with totally negative cerebrospinal fluid analysis after only 1 month of therapy.

In conclusion, in our case, after the diagnosis of LC, despite the presence of neurological symptoms that are generally linked to a bad prognosis and the only active treatment with TDM1, without locoregional treatments, the patient is still alive after a fast complete response on LC. The patient has experienced 16 months of overall survival, the absence of neurological symptoms, and good general conditions (PS 1 sec ECOG).

As such, it is important to carry out further research on LC, as well as the pathological and clinical features of BC subtypes, to lead a more personalized diagnostic and therapeutic program for these patients.

## Data Availability

The datasets generated and/or analyzed during the current study are available from the corresponding author upon reasonable request.
